# The Synthesis of a Large Stokes-Shift Dye and Intercalation into the Nanochannels of Zeolite L

**DOI:** 10.3390/ma17225669

**Published:** 2024-11-20

**Authors:** Fabian Walther, Achim Ecker, Dominik Brühwiler, Marc Bornand

**Affiliations:** Institute of Chemistry and Biotechnology, ZHAW Zurich University of Applied Sciences, CH-8820 Wädenswil, Switzerlandecke@zhaw.ch (A.E.);

**Keywords:** Stokes shift, fluorescence, zeolite, nanochannel, host–guest, intercalation

## Abstract

A host–guest-based fluorescent composite with a large Stokes shift was synthesized by intercalating 2,2′-(thiophene-2,5-diyl)bis(benzo[d]oxazol-6-amine) (BBTA) into the nanochannels of zeolite L (ZL) and sealing the pores with (3-aminopropyl)triethoxysilane (APTES). To confirm the orientation of the amino groups in BBTA, a single crystal of 2,5-bis(6-nitrobenzo[d]oxazol-2-yl)thiophene (BBTN) was grown and examined by X-ray crystallography. The evidence of successful intercalation of BBTA into the nanochannels of ZL was provided by fluorescence spectrometry, gas sorption and fluorescence microscopy. BBTA showed a Stokes shift of 6641 cm^−1^ (157 nm) in ethanol and 4611 cm^−1^ (93 nm) in toluene. The BBTA-ZL composite (BBTA-ZL-s) showed a Stokes shift of 5677 cm^−1^ (123 nm) in toluene, and 5450 cm^−1^ (124 nm) in ethanol. In addition, the degree of loading was determined and stability against leaching was confirmed. We report the synthesis of this novel composite dye material with potential applications where free dyes are not applicable and which retains a large Stokes shift, independent of its chemical environment.

## 1. Introduction

Composites obtained by the intercalation of chromophores and fluorophores into the nanochannels of zeolite L (ZL) have generated interest in various fields, including the development of novel colorants [1], photonic antenna systems [2], or fluorescent labels for optical imaging [3]. The supramolecular organization caused by the strong confinement of the guest molecules in the channels of ZL leads to anisotropic properties [4] and systems capable of efficient energy transfer [5]. The chromophores and fluorophores located in the ZL channels are shielded by the ZL framework. Consequently, the photophysical properties of the respective composites are typically independent of the surrounding medium [6]. Furthermore, the guest molecules are protected from chemical attack [1].

For light-harvesting applications such as luminescent solar concentrators [7,8,9], high concentrations of fluorophores are required, leading to self-absorption due to a non-negligible overlap between the absorption and emission spectrum [10]. Similarly, fluorophore-based probes or labels with a small Stokes shift often feature suboptimal signal-to-noise ratio and self-quenching. Fluorophores with large Stokes shifts have been emerging as a possible solution for these issues [11,12,13,14]. Based on the fact that ZL crystals can be synthesized in a large range of sizes and shapes [15,16,17,18], the intercalation of dyes with a large Stokes shift into the channels of ZL opens possibilities to obtain novel large Stokes shift fluorescent materials with a predetermined particle size, shape, and surface chemistry, as well as with anisotropic properties.

This work describes the synthesis, intercalation, and characterization of 2,2′-(thiophene-2,5-diyl)bis(benzo[d]oxazol-6-amine) (BBTA). The synthesis of BBTA starting from 2,5-bis(6-nitrobenzo[d]oxazol-2-yl)thiophene (BBTN) reported by Gao et al. [19] was optimized. Crystals suitable for X-ray diffraction could be obtained and the configuration and conformation of BBTN in the solid state was elucidated.

## 2. Materials and Methods

### 2.1. Chemicals

All experiments were carried out with the following commercially available reagents and solvents, which were used without further purification: 2,5-bis(benzo[d]oxazol-2-yl)thiophene (BBT, 97%, BOC-Sciences, Shirley, NY, USA), (3-aminopropyl)triethoxysilane (APTES, 99%, Sigma-Aldrich, Buchs, Switzerland), Chloroform-d (CDCl_3_, 99.8 %, Apollo, New York, NY, USA), dichloromethane (DCM, 99.8 %, VWR Chemicals, Schlieren, Switzerland), d_6_-DMSO (99.8%, Apollo), ethanol (99.95%, VWR Chemicals), ethyl acetate (99.5%, Honeywell, Charlotte, NC, USA), hydrochloric acid (37%, Sigma-Aldrich), nitric acid (65%, Roth AG, Solothurn, Switzerland), potassium nitrate (99%, Sigma-Aldrich), sodium hydroxide (98%, Sigma-Aldrich), sulphuric acid (98%, Roth AG), stannous chloride dihydrate (Reag. Ph. Eur., Merck, Rahway, NJ, USA), zeolite L (ZL, HSZ-500, Tosoh Corporation, Tokyo, Japan).

### 2.2. Synthesis of BBTN and BBTA

**Figure 1:**
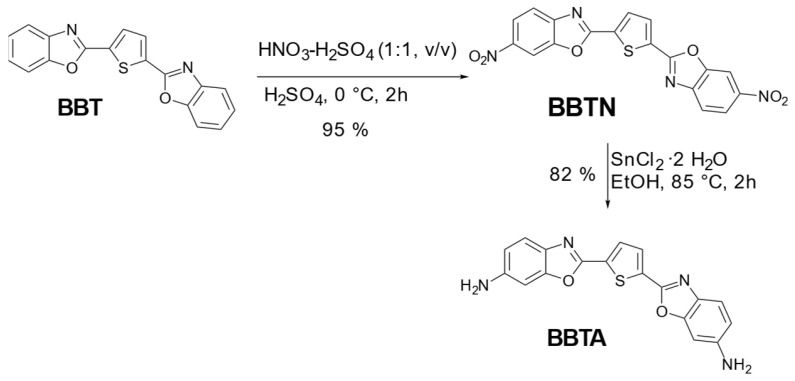
Reaction scheme for the production of BBTA starting from BBT via BBTN. BBT is first nitrated with a nitrating acid and then the nitro groups are reduced.

The reaction scheme for the synthesis of BBTN and BBTA is shown in Figure 1.

A solution of 2,5-bis(benzo[d]oxazol-2-yl)thiophene (BBT, 10.064 g, 31.61 mmol) in sulphuric acid 98% (50 mL) was prepared at 0 °C in a round-bottom flask (250 mL), which was equipped with a thermometer and a magnetic stirring bar. After 10 min the nitrating acid mixture (nitric acid–sulfuric acid 1:1 *v*/*v*, 10 mL) was added dropwise over 35 min. The solution was always kept below 8 °C. The reaction mixture changed color from light yellow to dark orange within a few minutes while dosing. The reaction mixture was stirred for 2 h at 0 °C. After the completion of the reaction (monitored by thin-layer chromatography), the reaction mixture was poured into ice water (500 mL), which was stirred with a magnetic stirring bar. A yellow solid precipitated. Ethyl acetate (50 mL) was added to the suspension and allowed to stir for 5 min. The suspension was filtered with a glass frit and reduced pressure. The filter residue was dispersed with water (5 × 100 mL) and the product was dried with a rotary evaporator under reduced pressure at a bath temperature of 45 °C to constant mass (12 h). No purification was required to yield BBTN (12.31 g, 95%) as a yellow solid. *R*_F_: 0.5 (cyclohexane/ethyl acetate 1:1); ^1^H-NMR (500 MHz, CDCl_3_): *δ* = 8.52 (d, *J* = 2.1 Hz, 2H), 8.37 (dd, *J* = 8.8, 2.1 Hz, 2H), 8.09 (s, 2H), 7.89 (d, *J* = 8.8 Hz, 2H) ppm; ^13^C-NMR (125 MHz, CDCl_3_): *δ* = 161.70, 149.83, 147.07, 145.69, 133.58, 132.21, 121.38, 120.19, 107.36 ppm.

BBTN (308 mg, 0.754 mmol) was dissolved in ethanol (6 mL) in a 2-neck round-bottom flask (25 mL) which was equipped with a magnetic stirring bar at room temperature. Stannous chloride dihydrate (1.703 g, 7.55 mmol) was added and the reaction mixture was heated to 85 °C, turning from a yellow to a dark orange solution at a bath temperature of 40 °C. The reaction mixture was refluxed for 2 h at 85 °C, then placed in water (100 mL) and adjusted to acidic pH with hydrochloric acid 37% (1 mL). A clear orange solution was obtained. Subsequently, the solution was adjusted to basic pH with sodium hydroxide solution 30% (3 mL). A solid precipitated at pH 5, which made the solution difficult to stir with a magnetic stirring bar. After 3 min the solid dissolved again. At pH 9 a dark red suspension was obtained. This suspension was filtered through a glass frit and washed with water (3 × 20 mL) to neutral pH. The product was dried in a vacuum oven at 60 °C for 4 h, yielding BBTA (216 mg, 82%) as a dark red solid. *R*_F_: 0.16 (cyclohexane/ethyl acetate 1:1); ^1^H-NMR (500 MHz, d_6_-DMSO): *δ* = 7.90 (s, 2H), 7.48 (d, *J* = 8.5 Hz, 2H), 6.87 (d, *J* = 2.1 Hz, 2H), 6.74 (dd, *J* = 8.5, 2.1 Hz, 2H), 5.64 (s, 4H) ppm. ^13^C-NMR (125 MHz, d_6_-DMSO): *δ* = 154.65, 152.34, 149.13, 131.99, 131.97, 129.71, 120.27, 113.44, 94.41 ppm.

### 2.3. Synthesis of the BBTA-ZL Composite

BBTA (74 mg) was suspended in DCM (30 mL) in a single-neck round-bottom flask (100 mL) and ZL (3.71 g) was added. The suspension was treated for 1 h in an ultrasonic bath at room temperature. DCM was subsequently removed with a rotary evaporator at a bath temperature of 45 °C and a pressure of 650 mbar. After 15 min, the pressure was reduced to 60 mbar and maintained for 1 h. A large oval magnetic stirring bar was added to the round-bottom flask containing the ZL powder impregnated with BBTA. The impregnated ZL was dried for 1 h under stirring at a pressure of 13 mbar and a bath temperature of 160 °C. The vacuum was broken with nitrogen and the light orange powder was stirred for 26 h at 160 °C under static nitrogen atmosphere. The resulting orange powder was cooled to room temperature. Subsequently, the product was washed by dispersing the particles in DCM (10 × 10 mL). The particles and DCM (10 mL) were placed in a Falcon tube (50 mL) and vortexed for 1 min and sonicated for 1 min. The suspension was centrifuged at 9000 rpm for 10 min. The supernatant solution was decanted, and the washing process was repeated until the supernatant was colorless. The obtained particles were dried for 4 h in a vacuum drying oven at 40 °C to constant mass, yielding 3.2 g of BBTA-ZL as a slightly orange powder. For imaging by fluorescence microscopy, larger ZL crystals [15] were used.

To avoid the leaching of BBTA, the channels of BBTA-ZL were sealed. BBTA-ZL (100 mg) was suspended in ethyl acetate (1.5 mL) at room temperature in a GC-headspace vial. APTES (0.3 mL) was added in under 3 s. The GC-headspace vial was then sealed, and the reaction was stirred with a magnetic stirring bar for 2 h at room temperature. The oil bath was set to 80 °C and left at this temperature for 19 h. The suspension was allowed to cool to room temperature and washed with DCM (7 × 10 mL). For this purpose, the particles and DCM (10 mL) were placed in a Falcon tube (50 mL) and treated for 1 min on a vortex mixer and 1 min in the ultrasonic bath. The suspension was centrifuged at 9000 rpm for 10 min. The supernatant solution was decanted off and the washing process was repeated until the supernatant was colorless. The obtained particles were dried for 4 h in a vacuum oven at 40 °C to constant mass, yielding 80 mg of dark orange powder. The sealed BBTA-ZL was designated as BBTA-ZL-s.

### 2.4. Characterization

The reactions were monitored by thin-layer chromatography (TLC) using Silicycle silica gel 60 F254 plates (0.2 mm thickness, Merck). NMR spectra were recorded at 500 MHz (for ^1^H-NMR), 125 MHz (for ^13^C-NMR), in CDCl_3_ or d_6_-DMSO (Bruker Ascend TM500). Chemical shifts are reported as *δ* (ppm) downfield from tetramethylsilane (TMS) (*δ* = 0.00) using residual solvent signal as an internal standard: *δ* singlet 7.26 (^1^H), triplet 77.0 (^13^C). Fluorescence spectra were measured with a fluorescence spectrophotometer (Fluorolog-3, Horiba, Kyoto, Japan). Dark offset was used as correction for the measurement of the pure dye. An amount of approx. 1 mg of BBTA was dissolved in the respective solvent (1 mL). A volume of 0.2 mL of this solution was diluted with 2.8 mL of pure solvent. To measure BBTA-ZL composites, 4 mg of the composite in 1 mL of solvent was treated with an ultrasonic bath for 30 min. From this suspension, 0.25 mL was added to 2.75 mL of pure solvent. Fluorescence microscopy was performed with an Olympus BX60 microscope (Wallisellen, Switzerland).

The nitrogen sorption isotherms were measured at 77 K with a Quantachrome Autosorb iQ MP (Anton-Paar, Baden, Switzerland). All samples were vacuum degassed at 80 °C for 24 h prior to the sorption measurements. The total pore volume (V_tot_) was calculated from the amount of adsorbed nitrogen at a relative pressure of ca. 0.95.

To grow a single crystal, a saturated solution of BBTN in chloroform (3 mL) was prepared. The resulting suspension was filtered through cotton wool into an NMR tube filled with cyclohexane (3 mL). The NMR tube was sealed and stored at room temperature for 3 weeks. A suitable crystal was selected and measured on a SuperNova, Dual Cu at home/near, Atlas diffractometer. The crystal was kept at 160.15 K during data collection. Using Olex2 (version 1.3), the structure was solved with the XT structure solution program using Intrinsic Phasing and refined with the XL refinement package using Least Squares minimalization.

To test the stability against leaching, a supersaturated solution of potassium nitrate was prepared in a desiccator. For this purpose, 90 g of KNO_3_ was added to 60 g of water and placed in a desiccator for 48 h to reach saturation. The relative humidity was checked using a hygrometer. A relative humidity of 92% [20] was obtained. Approx. 250 mg of BBTA-ZL or BBTA-ZL-s was added to a flat Petri dish and distributed homogeneously. The samples were left in the dish for 24 h. The samples were then washed with DCM (10 mL) until a constant absorbance of the washing solution was achieved.

## 3. Results and Discussion

### 3.1. BBTN

**Figure 2:**
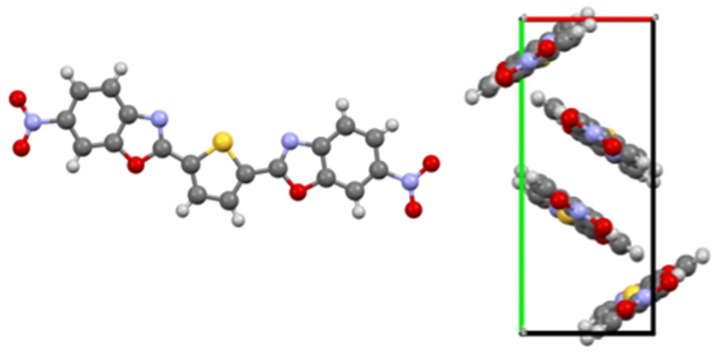
X-ray structure of BBTN (**left**) and the corresponding unit cell (**right**). Created with VESTA ver. 3.5.8 based on the cif-file deposited at CCDC with number 2252524.

The nitration of BBT could be reproduced according to the literature [19]. The exact constitution of the nitro groups could not be determined from the NMR data (^1^H, ^13^C, COSY, HMBC). Therefore, a single crystal was grown to determine the structure of BBTN by X-ray diffraction (Figure 2). The substitution pattern and the overall structure was confirmed. The nitro groups are in para position to the nitrogen in the oxazole. The absence of multiple nitration is due to the deactivation of the benzene ring by the nitro group and its strong negative mesomeric effect. BBTN in the single crystal features a similar spacing as graphite [21].

### 3.2. BBTA

Starting from BBTN, the nitro groups were reduced. Stannous chloride dihydrate, iron with acetic acid, and H_2_/Pd were tested as reducing agents. It was found that iron with acetic acid, as well as H_2_/Pd, led to complete conversion of the reactant after 72 h. No by-products were formed. In the case of stannous chloride dihydrate, complete conversion was already observed after 2 h. If the reaction time was increased, by-products formed. It is noteworthy that BBTA was not detectable on a TLC plate at 254 nm. It only became visible at 366 nm (Figure S1). The reason for this was that BBTA emitted the same wavelength as the fluorescent indicator on the TLC plate. On an uncoated TLC plate, BBTA was visible at 254 nm (Figure S2). For this reason, we recommend detection at 366 nm. The comparison of the NMR with data reported in the literature [19] showed some inconsistencies. In the ^1^H-NMR, large differences in the coupling constants were obtained in some cases. Furthermore, Gao et al. [19] described eight signals in the ^13^C-NMR, whereas nine signals should be obtained, as shown here (the spectra can be found in the Supporting Information).

**Figure 3:**
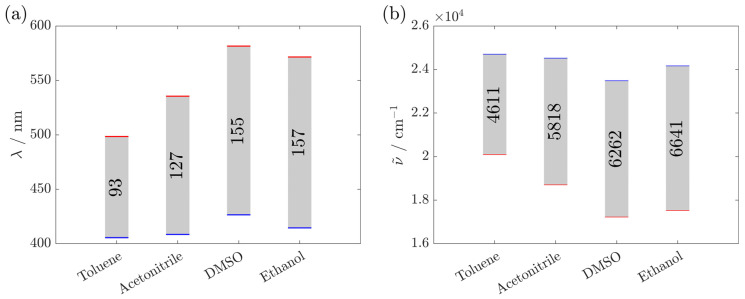
Stokes shift in BBTA (66 µg·mL^−1^) in (**a**) wavelength and (**b**) wavenumber in different solvents. The maxima, blue for excitation and red for emission, are highlighted. The Stokes shift is shown as a gray bar. The exact excitation and emission wavelength maxima are listed in Tables S1 and S2.

**Figure 4:**
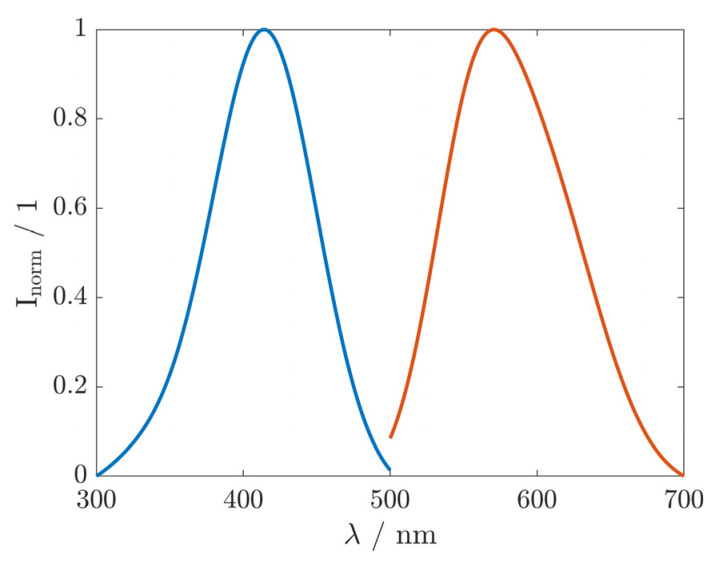
Normalized (I_norm_) excitation spectrum (blue) and the emission spectrum (orange) of BBTA (66 µg·mL^−1^) in ethanol.

The Stokes shifts obtained for BBTA in various solvents are summarized in Figure 3. The emission of BBTA was shifted to longer wavelengths when the polarity of the solvent was increased from toluene, acetonitrile to DMSO according to Reichardt’s scale [22,23]. However, for ethanol, which exhibits a lower dipole moment than DMSO, a more pronounced Stokes shift was observed. This indicates that the fluorescence of BBTA is influenced by both the polarity and the hydrogen bonding ability of the solvent. These large shifts in emission to longer wavelengths with increasing polarity and hydrogen bonding ability of the solvent suggest that BBTA has significant polarity in the excited state, making it more susceptible to the influence of polar or protic solvents [19,24,25]. Furthermore, the large Stokes shift suggests a structural change upon excitation [26]. This was confirmed with state-specific TD-DFT calculations of BBTA, which yielded substantial differences in the ground and excited state dipole moments [24]. In the fluorescence spectrum, no fine structure was observed in any of the investigated solvents (shown exemplarily in ethanol, Figure 4), indicating a molecule that is not completely rigid in the excited state [27].

### 3.3. BBTA-ZL

**Figure 5:**
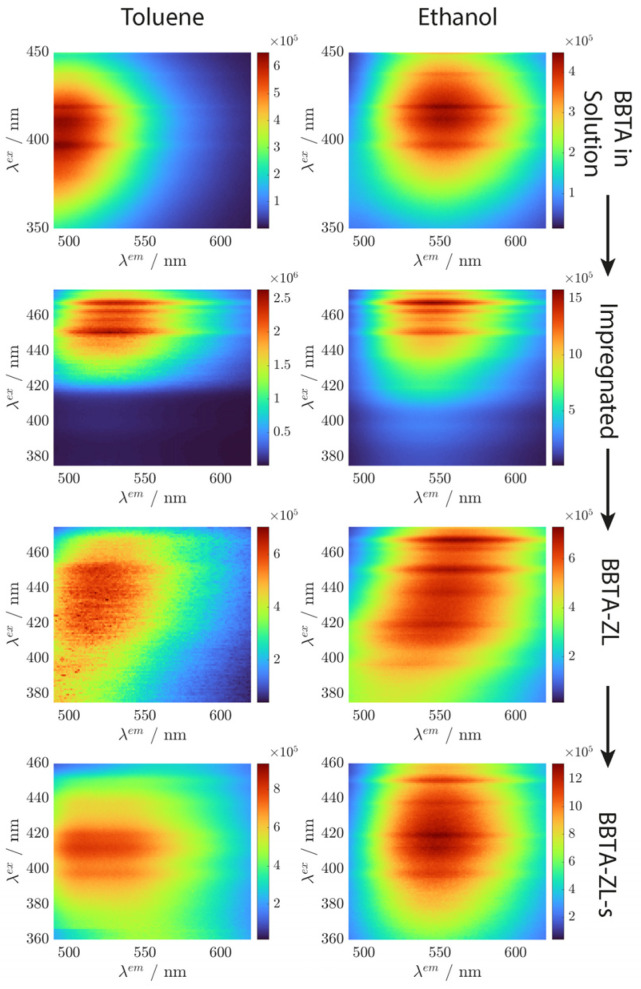
Three-dimensional fluorescence spectra measured in toluene (**left column**) and in ethanol (**right column**). From top to bottom: BBTA in solution (66 µg·mL^−1^), BBTA on the external surface of ZL (Impregnated, 333 µg·mL^−1^), BBTA-ZL (333 µg·mL^−1^), and BBTA-ZL-s (333 µg·mL^−1^). The excitation wavelength is plotted on the *y*-axis and the emission wavelength is plotted on the *x*-axis.

It first must be shown that BBTA is in fact located in the nanochannels of ZL as opposed to being adsorbed on the external zeolite surface. Successful intercalation would lead to an almost identical 3D fluorescence spectrum of BBTA-ZL in solvents of different polarity because the intercalated BBTA is shielded from the surrounding solvent by the zeolite framework. The respective spectra are shown in Figure 5.

As expected, the spectra of BBTA dissolved in toluene and in ethanol are distinctively different. After the impregnation of the ZL crystals with BBTA, the excitation wavelength shifted to longer wavelengths. It can be assumed that impregnation leads to the deposition of BBTA aggregates on the external ZL surface, which are still exposed to the surrounding solvent. BBTA-ZL obtained after intercalation showed a rather undefined 3D spectrum. This can be attributed to some leaching of BBTA, which results in the presence of BBTA in the solution and on the external ZL surface, in addition to the BBTA located in the ZL nanochannels. To prevent leaching, the nanochannels of ZL were sealed with APTES [2,28]. As a result, the solvatochromic effect observed in the solution was significantly reduced. This is a first indication of successful intercalation. The general applicability of this concept was evaluated by preparing and intercalating another molecule with a large Stokes shift. The results are provided in the Supporting Information.

Considering the structure of BBTA and the acidic conditions in the ZL nanochannels [29], it is surprising that the channel entrances had to be sealed to prevent leaching. It can be assumed that the amino group of BBTA is protonated in ZL leading to electrostatic interactions with the negatively charged zeolite framework. A possible explanation is that the mobility of BBTA was too low during intercalation. It has been previously observed that slow intrachannel diffusion might lead to an accumulation of the guest species on sites close to the channel entrances [30]. BBTA lacks the alkyl substituents that were found to be crucial for intrachannel mobility [2].

**Figure 6:**
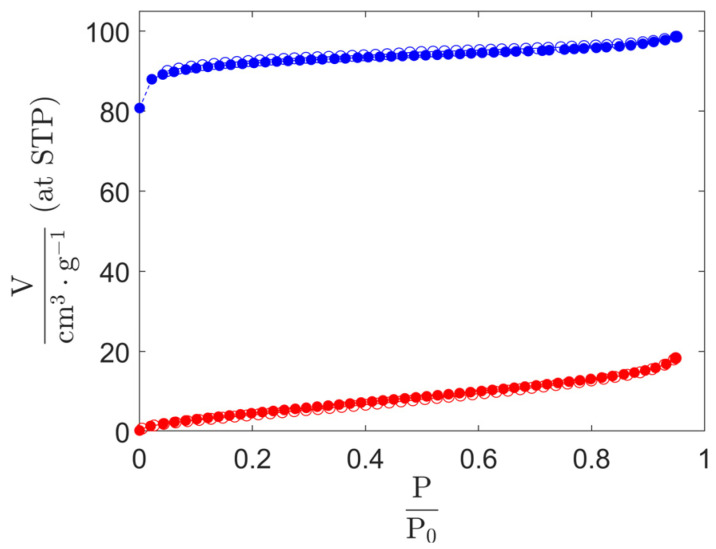
Nitrogen sorption isotherms at 77 K of pristine ZL (blue) and BBTA-ZL (red). The adsorption isotherms are shown as solid circles and the desorption isotherms as hollow circles. Adsorption and desorption branches coincide.

To confirm the above results, the materials were further analyzed by nitrogen sorption (Figure 6). The incorporation of BBTA molecules within the ZL channels results in a substantial decrease in the accessible pore volume. Figure 6 illustrates the nitrogen isotherm of ZL and BBTA-ZL. As predicted, ZL presents a type I(a) isotherm, typically observed for microporous materials possessing predominantly narrow micropores [31]. The capacity for nitrogen adsorption is markedly reduced following the intercalation of BBTA. A total pore volume of 0.153 cm^3^·g^−1^ was determined for the pristine ZL sample, whereas BBTA-ZL exhibited a total pore volume of only 0.028 cm^3^·g^−1^. This is indicative of the intense spatial confinement in BBTA-ZL, which, in conjunction with the one-dimensionality of the ZL channel system, restricts diffusion and thus protects the intercalated BBTA molecules from chemical attack. The sealing of BBTA-ZL with APTES led to a further reduction in the total pore volume (V_tot_ = 0.013 cm^3^·g^−1^).

**Figure 7:**
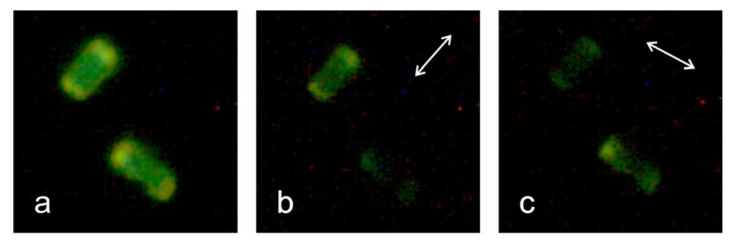
Fluorescence microscopy images of BBTA-ZL. The images show two crystals with a length of ca. 6.5 µm. For both crystals, the c-axis corresponds to the longer axis of the crystals. Image (**a**) was taken without a polarizer. The double arrows in images (**b**,**c**) indicate the direction of the polarizer.

Geometrical confinement leads to a defined orientation of the guest molecules in the ZL channels. In the case of fluorescent molecules, the orientation can be observed by polarized fluorescence microscopy. For long-stretched molecules, alignment along the channel axis (c-axis) of the ZL crystals has been observed, whereas smaller molecules tend to adopt a tilted orientation [4,32]. No angle dependence of the fluorescence can be observed if the electronic transition dipole moment of the guest molecule is aligned along the magic angle [4]. Figure 7 shows that in the case of BBTA-ZL, the fluorescence is largely quenched when the polarizer is set at 90° relative to the c-axis of the ZL crystals. This indicates that BBTA is preferentially aligned along the channel axis.

### 3.4. BBTA-ZL-s

**Figure 8:**
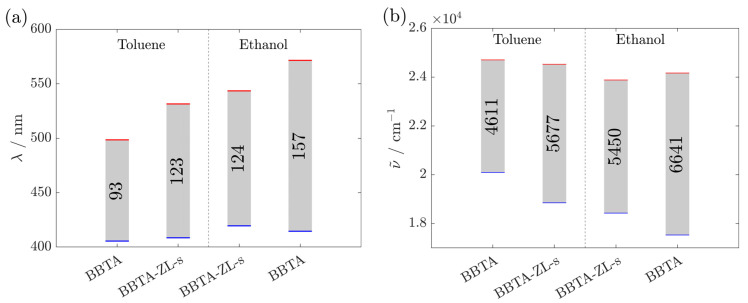
Stokes shift in BBTA (66 µg·mL^−1^) and BBTA-ZL-s (333 µg·mL^−1^) in (**a**) wavelength and (**b**) wavenumber in toluene and ethanol. The maxima, blue for excitation and red for emission, are highlighted. The Stokes shift is shown as a gray bar. The exact excitation and emission wavelength maxima are listed in Tables S3 and S4.

To increase stability against leaching, sealing of the ZL channels with APTES is advantageous. The resulting sample, designated as BBTA-ZL-s, features a constant Stokes shift independent of the surrounding solvent (Figure 8). Interestingly, the Stokes shift in BBTA in ZL is slightly smaller than for BBTA dissolved in ethanol, although the ZL channels are a highly polar environment [29]. Restrictions in terms of conformational states could be the reason for the smaller Stokes shift in BBTA in ZL compared to BBTA dissolved in ethanol [33]. The slightly deviating excitation and emission maxima can be explained by the different light scattering properties of suspensions of ZL in toluene and ethanol.

To determine the stability against leaching, the loading of the ZL crystals with BBTA was calculated by quantifying the amount of BBTA in the washing solutions after the intercalation step. A mass percentage of 1.39% BBTA (relative to the mass of ZL) was obtained. BBTA-ZL and BBTA-ZL-s were then exposed to a relative humidity of 92% for 24 h at room temperature. For BBTA-ZL, the loading level decreased to 1.25%. BBTA-ZL-s showed higher stability against leaching, with the loading level decreasing to 1.35% after the same time period.

## 4. Conclusions

BBTA was synthesized, characterized, and intercalated into the nanochannels of ZL. A Stokes shift is typically considered large if it is greater than 80 nm [19] or 100 nm [34], respectively. The large Stokes shift in BBTA is preserved after intercalation and sealing of the ZL channels. The resulting BBTA-ZL-s composite is a particle-shaped fluorophore with intriguing properties. Its large Stokes shift is essentially independent of the surrounding medium, as BBTA is shielded by the zeolite framework. Furthermore, due to the alignment of BBTA in the ZL channels, the composite shows polarized fluorescence. The size and shape of the composite can be adjusted over a wide range (several nm to tenths of µm and from disks to needles) by making use of established procedures for the synthesis of ZL crystals [15,16,17,18]. Furthermore, the external surface of the ZL crystals allows for versatile functionalization with targeting ligands, antibodies, or other biomolecules, for example. When the nanochannels are sealed, the dye remains within the zeolite, potentially rendering the resulting particles non-toxic [35]. The sum of these properties opens possibilities for the use of these composites in applications such as biological imaging [36,37], optical devices [38], or security printing [39].

## Data Availability

The original contributions presented in the study are included in the article and Supplementary Materials, further inquiries can be directed to the corresponding author.

## Supplementary Materials

The following supporting information can be downloaded at: https://www.mdpi.com/article/10.3390/ma17225669/s1, Figure S1: Left: TLC, from left to right: different amounts of BBTA. Left, at 254 nm. Right, same TLC but recorded at 366 nm. The solvent used was EtOAc:cHex (1:1); Figure S2: 3 µL of BBTA solution (1 mg·mL^−1^) was applied to a TLC plate with fluorescence indicator (top row) and to a TLC plate without fluorescence indicator (bottom row). On the TLC plate with fluorescence indicator, BBTA is not visible at 254 nm, because it emits the same color as the indicator. On an unlabeled TLC plate, BBTA is therefore visible at 254 nm. Both plates were imaged at 366 nm; Figure S3: ^1^H-NMR of BBTN in CDCl_3_, Figure S4: ^13^C-NMR of BBTN in CDCl_3_; Figure S5: ^1^H-NMR of BBTA in d_6_-DMSO; Figure S6: ^13^C-NMR of BBTA in d_6_-DMSO; Figure S7: Zoom of the ^13^C-NMR of BBTA in d_6_-DMSO from 132 to 130.5 ppm; Figure S8: Stokes shift of 7 (66 µg·mL^−1^) in (a) wavelength and (b) wavenumber in different solvents. The maxima, blue for excitation and red for emission, are highlighted. The Stokes shift is shown as a gray bar. The exact excitation and emission wavelength maxima are listed in Tables S5 and S6; Figure S9: 3D fluorescence spectra measured in toluene (left column) and in ethanol (right column). From top to bottom: 7 in solution (66 µg·mL^−1^), 7 on the external surface of ZL (Impregnated, 333 µg·mL^−1^), and 7-ZL-s (333 µg·mL^−1^). The excitation wavelength is plotted on the *y*-axis and the emission wavelength is plotted on the *x*-axis; Figure S10: Stokes shift of 7 (66 µg·mL^−1^) and 7-ZL-s (333 µg·mL^−1^) in (a) wavelength and (b) wavenumber in toluene and ethanol. The maxima, blue for excitation and red for emission, are highlighted. The Stokes shift is shown as a gray bar. The exact excitation and emission wavelength maxima are listed in Tables S7 and S8; Figure S11: ^1^H-NMR of 5 in CDCl_3_; Figure S12: ^13^C-NMR of 5 in CDCl_3_; Figure S13: ^1^H-NMR of 7 in d_6_-DMSO; Figure S14: ^13^C-NMR of 7 in d_6_-DMSO; Table S1: Data for Figure 3a. BBTA in different solvents with the excitation and emission maximum and the corresponding Stokes shift; Table S2: Data for Figure 3b. BBTA in different solvents with the excitation and emission maximum and the corresponding Stokes shift; Table S3: Data for Figure 8a. BBTA and BBTA-ZL-s in different solvents with the excitation and emission maximum and the corresponding Stokes shift; Table S4: Data for Figure 8b. BBTA and BBTA-ZL-s in different solvents with the excitation and emission maximum and the corresponding Stokes shift; Table S5: Data for Figure S8a. Molecule 7 in different solvents with the excitation and emission maximum and the corresponding Stokes shift; Table S6: Data for Figure S8b. Molecule 7 in different solvents with the excitation and emission maximum and the corresponding Stokes shift; Table S7: Data for Figure S10a. 7 and 7-ZL-s in different solvents with the excitation and emission maximum and the corresponding Stokes shift; Table S8: Data for Figure S10b. 7 and 7-ZL-s in different solvents with the excitation and emission maximum and the corresponding Stokes shift. Reference [40] is cited in the Supplementary Materials.

## References

[1] Woodtli P., Giger S., Müller P., Sägesser L., Zucchetto N., Reber M.J., Ecker A., Brühwiler D. (2018). Indigo in the Nanochannels of Zeolite L: Towards a New Type of Colorant. Dye. Pigment..

[2] Cao P., Khorev O., Devaux A., Sägesser L., Kunzmann A., Ecker A., Häner R., Brühwiler D., Calzaferri G., Belser P. (2016). Supramolecular Organization of Dye Molecules in Zeolite L Channels: Synthesis, Properties, and Composite Materials. Chem. Eur. J..

[3] Strassert C.A., Otter M., Albuquerque R.Q., Höne A., Vida Y., Maier B., De Cola L. (2009). Photoactive Hybrid Nanomaterial for Targeting, Labeling, and Killing Antibiotic-Resistant Bacteria. Angew. Chem. Int. Ed..

[4] Brühwiler D., Calzaferri G., Torres T., Ramm J.H., Gartmann N., Dieu L.-Q., López-Duarte I., Martínez-Díaz M.V. (2009). Nanochannels for Supramolecular Organization of Luminescent Guests. J. Mater. Chem..

[5] Devaux A., Calzaferri G., Belser P., Cao P., Brühwiler D., Kunzmann A. (2014). Efficient and Robust Host–Guest Antenna Composite for Light Harvesting. Chem. Mater..

[6] Calzaferri G., Brühwiler D., Megelski S., Pfenniger M., Pauchard M., Hennessy B., Maas H., Devaux A., Graf U. (2000). Playing with Dye Molecules at the Inner and Outer Surface of Zeolite L. Solid State Sci..

[7] Weber W.H., Lambe J. (1976). Luminescent Greenhouse Collector for Solar Radiation. Appl. Opt..

[8] Batchelder J.S., Zewail A.H., Cole T. (1979). Luminescent Solar Concentrators. 1: Theory of Operation and Techniques for Performance Evaluation. Appl. Opt..

[9] Goetzberger A., Greubel W. (1977). Solar Energy Conversion with Fluorescent Collectors. Appl. Phys..

[10] Dienel T., Bauer C., Dolamic I., Brühwiler D. (2010). Spectral-Based Analysis of Thin Film Luminescent Solar Concentrators. Sol. Energy.

[11] Liu H., Jiang G., Ke G., Ren T., Yuan L. (2024). Organic Fluorophores with Large Stokes Shift for Bioimaging and Biosensing. ChemPhotoChem.

[12] Horváth P., Šebej P., Šolomek T., Klán P. (2015). Small-Molecule Fluorophores with Large Stokes Shifts: 9-Iminopyronin Analogues as Clickable Tags. J. Org. Chem..

[13] Lehnert M., Kipf E., Schlenker F., Borst N., Zengerle R., Von Stetten F. (2018). Fluorescence Signal-to-Noise Optimisation for Real-Time PCR Using Universal Reporter Oligonucleotides. Anal. Methods.

[14] Yue Y., Zhao T., Xu Z., Chi W., Chai X., Ai J., Zhang J., Huo F., Strongin R.M., Yin C. (2023). Enlarging the Stokes Shift by Weakening the *π* -Conjugation of Cyanines for High Signal-to-Noise Ratiometric Imaging. Adv. Sci..

[15] Ruiz A.Z., Brühwiler D., Ban T., Calzaferri G. (2005). Synthesis of Zeolite L. Tuning Size and Morphology. Monatshefte Chem. Chem. Mon..

[16] Gomez A.G., de Silveira G., Doan H., Cheng C.-H. (2011). A Facile Method to Tune Zeolite L Crystals with Low Aspect Ratio. Chem. Commun..

[17] Ban T., Saito H., Naito M., Ohya Y., Takahashi Y. (2007). Synthesis of Zeolite L Crystals with Different Shapes. J. Porous Mater..

[18] Tsapatsis M., Lovallo M., Okubo T., Davis M.E., Sadakata M. (1995). Characterization of Zeolite L Nanoclusters. Chem. Mater..

[19] Gao Z., Hao Y., Zheng M., Chen Y. (2017). A Fluorescent Dye with Large Stokes Shift and High Stability: Synthesis and Application to Live Cell Imaging. RSC Adv..

[20] Kutzelnigg A., Königsheim F. (1963). Die Einstellung Der Relativen Feuchte Mit Hilfe von Gesättigten Salzlösungen. Mater. Corros..

[21] Robin M., Faure R., Périchaud A., Galy J.-P. (2002). Synthesis of new tetracycle fused acridine analogues bearing oxazole ring. Synth. Commun..

[22] Reichardt C. (1979). Empirical Parameters of Solvent Polarity as Linear Free-Energy Relationships. Angew. Chem. Int. Ed. Engl..

[23] Reichardt C. (1994). Solvatochromic Dyes as Solvent Polarity Indicators. Chem. Rev..

[24] Su Y., Ren H., Li X. (2019). Novel Nonequilibrium Solvation Theory for Calculating the Solvatochromic Stokes Shift by State-Specific TD-DFT. Chem. Phys. Lett..

[25] Fung S.Y., Duhamel J., Chen P. (2006). Solvent Effect on the Photophysical Properties of the Anticancer Agent Ellipticine. J. Phys. Chem. A.

[26] Divac V.M., Šakić D., Weitner T., Gabričević M. (2019). Solvent Effects on the Absorption and Fluorescence Spectra of Zaleplon: Determination of Ground and Excited State Dipole Moments. Spectrochim. Acta A Mol. Biomol. Spectrosc..

[27] Turro N.J. (1991). Modern Molecular Photochemistry.

[28] Lustenberger S., Brühwiler D. (2024). Sealing of Zeolite L Channels with Ethoxysilanes: Influence of Molecular Structure and Thermal Treatment. ChemistrySelect.

[29] Albuquerque R.Q., Calzaferri G. (2007). Proton Activity Inside the Channels of Zeolite L. Chem. Eur. J..

[30] Pfenniger M., Calzaferri G. (2000). Intrazeolite Diffusion Kinetics of Dye Molecules in the Nanochannels of Zeolite L, Monitored by Energy Transfer. ChemPhysChem.

[31] Thommes M., Kaneko K., Neimark A.V., Olivier J.P., Rodriguez-Reinoso F., Rouquerol J., Sing K.S.W. (2015). Physisorption of Gases, with Special Reference to the Evaluation of Surface Area and Pore Size Distribution (IUPAC Technical Report). Pure Appl. Chem..

[32] Calzaferri G., Huber S., Maas H., Minkowski C. (2003). Host–Guest Antenna Materials. Angew. Chem. Int. Ed..

[33] Más-Montoya M., García Alcaraz A., Espinosa Ferao A., Bautista D., Curiel D. (2023). Insight into the Stokes Shift, Divergent Solvatochromism and Aggregation-Induced Emission of Boron Complexes with Locked and Unlocked Benzophenanthridine Ligands. Dye. Pigment..

[34] Mohd Yusof Chan N.N., Idris A., Zainal Abidin Z.H., Tajuddin H.A., Abdullah Z. (2021). White Light Employing Luminescent Engineered Large (Mega) Stokes Shift Molecules: A Review. RSC Adv..

[35] Khodadadi Yazdi M., Zarrintaj P., Hosseiniamoli H., Mashhadzadeh A.H., Saeb M.R., Ramsey J.D., Ganjali M.R., Mozafari M. (2020). Zeolites for Theranostic Applications. J. Mater. Chem. B.

[36] Ma Y., Niu J., Liang X., Wang L., Zhang Y., Lv H., Wang T., Wang J., Zhang X., Xu S. (2023). In-Situ Gastritis Diagnosis by an Oral-Administration NIR Fluorescent Probe with a Large Stokes Shift and High Signal-to-Noise Ratio. Chem. Eng. J..

[37] Zhang J., Chen R., Zhu Z., Adachi C., Zhang X., Lee C.-S. (2015). Highly Stable Near-Infrared Fluorescent Organic Nanoparticles with a Large Stokes Shift for Noninvasive Long-Term Cellular Imaging. ACS Appl. Mater. Interfaces.

[38] Zhao Y., Chen K., Zhu L., Huang Q. (2023). Data-Driven Machine Learning Models for Quick Prediction of the Stokes Shift of Organic Fluorescent Materials. Dye. Pigment..

[39] Zhang J., Li R., Bei Y., Xu X.-D., Kang W. (2023). Design of a Large Stokes Shift Ratiometric Fluorescent Sensor with Hypochlorite Detection towards the Potential Application as Invisible Security Ink. Spectrochim. Acta A Mol. Biomol. Spectrosc..

[40] Mandal M., Chatterjee T., Das A., Mandal S., Sen A., Ta M., Mandal P.K. (2019). Meta-Fluors—A Unique Way to Create a 200 Da Ultrasmall Fluorophore Emitting in Red with Intense Stokes/Solvatochromic Shift: Imaging Subcellular Nanopolarity in Live Stem Cells. J. Phys. Chem. C.

