# Supplementary material for: The Synthesis of a Large Stokes-Shift Dye and Intercalation into the Nanochannels of Zeolite L
Source: Materials (Basel). 2024 Nov 20;17(22):5669. doi: 10.3390/ma17225669 (PMC11595820; doi:10.3390/ma17225669)
Supplement: Supplementary file 1 [file materials-17-05669-s001.zip › V16_Publikation_Stokes-Shift_ESI_MDPI_final.pdf]

Supporting Information

Synthesis of a large Stokes shift dye and intercalation into the nanochannels of zeolite L

Fabian Walther, Achim Ecker, Dominik Brühwiler, Marc Bornand*

* Corresponding Author. E-mail: marc.bornand@zhaw.ch (M.Bornand)

Institute of Chemistry and Biotechnology, Zürich University of Applied Sciences (ZHAW), CH- 8820 Wädenswil, Switzerland

Contents

1. TLC of BBTA
2. Stokes shift data of BBTA and BBTA-ZL composites
3. NMR of the products in the synthesis of BBTA
4. Synthesis of molecule 7
5. Stokes shift of 7 in different solvents
6. 3D fluorescence spectra of 7 in solution, impregnated and intercalated
7. Stokes shift of 7 after intercalation
8. NMR of the products in the synthesis of 7

1. TLC of BBTA

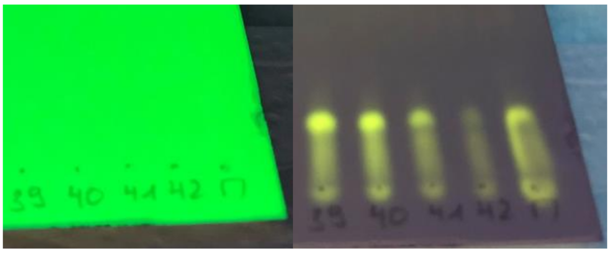

Figure S1: Left: TLC, from left to right: different amounts of BBTA. Left, at 254 nm. Right, same TLC but recorded at 366 nm. The solvent used was EtOAc:cHex (1:1).

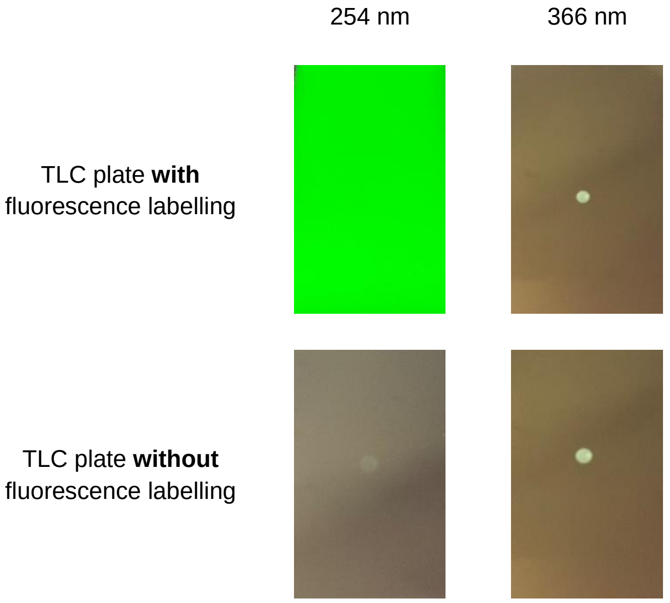

Figure S2: 3 µL of BBTA solution (1 mg·mL^{-1}) was applied to a TLC plate with fluorescence indicator (top row) and to a TLC plate without fluorescence indicator (bottom row). On the TLC plate with fluorescence indicator, BBTA is not visible at 254 nm, because it emits the same color as the indicator. On an unlabelled TLC plate, BBTA is therefore visible at 254 nm. Both plates were imaged at 366 nm.

2. Stokes shift data of BBTA and BBTA-ZL composites

Table S1: Data for Figure 3a. BBTA in different solvents with the excitation and emission maximum and the corresponding Stokes shift.

Solvent	λ_{ex}^{max} / nm	λ_{em}^{max} / nm	Stokes shift / nm
Toluene	405	498	93
Acetonitrile	408	535	127
DMSO	426	581	155
Ethanol	414	571	157

Table S2: Data for Figure 3b. BBTA in different solvents with the excitation and emission maximum and the corresponding Stokes shift.

Solvent	$\tilde{\nu}_{ex}^{max}$ / cm^{-1}	$\tilde{\nu}_{em}^{max}$ / cm^{-1}	Stokes shift / cm^{-1}
Toluene	24691	20080	4611
Acetonitrile	24510	18692	5818
DMSO	23474	17212	6262
Ethanol	24155	17513	6641

Table S3: Data for Figure 9a. BBTA and BBTA-ZL-s in different solvents with the excitation and emission maximum and the corresponding Stokes shift.

Analyte	Solvent	λ_{ex}^{max} / nm	λ_{em}^{max} / nm	Stokes shift / nm
BBTA	Toluene	405	498	93
BBTA-ZL-s	Toluene	408	531	123
BBTA-ZL-s	Ethanol	419	543	124
BBTA	Ethanol	414	571	157

Table S4: Data for Figure 9b. BBTA and BBTA-ZL-s in different solvents with the excitation and emission maximum and the corresponding Stokes shift.

Analyte	Solvent	$\tilde{\nu}_{ex}^{max}$ / cm^{-1}	$\tilde{\nu}_{em}^{max}$ / cm^{-1}	Stokes shift / cm^{-1}
BBTA	Toluene	24691	20080	4611
BBTA-ZL-s	Toluene	24510	18832	5677
BBTA-ZL-s	Ethanol	23866	18416	5450
BBTA	Ethanol	24155	17513	6641

3. NMR of the products in the synthesis of BBTA

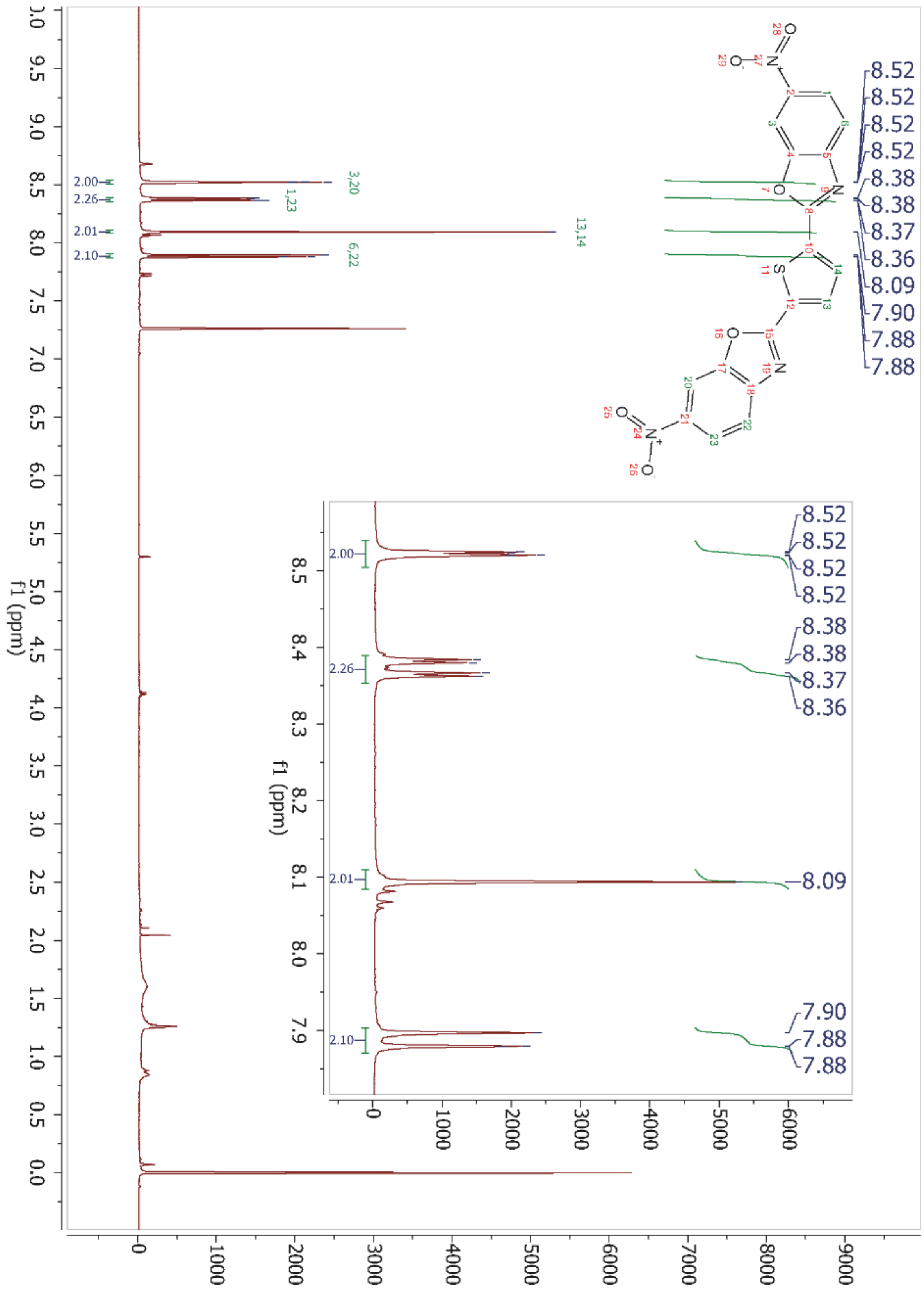

Figure S3: ^{1}H-NMR of BBTN in $CDCl_3$

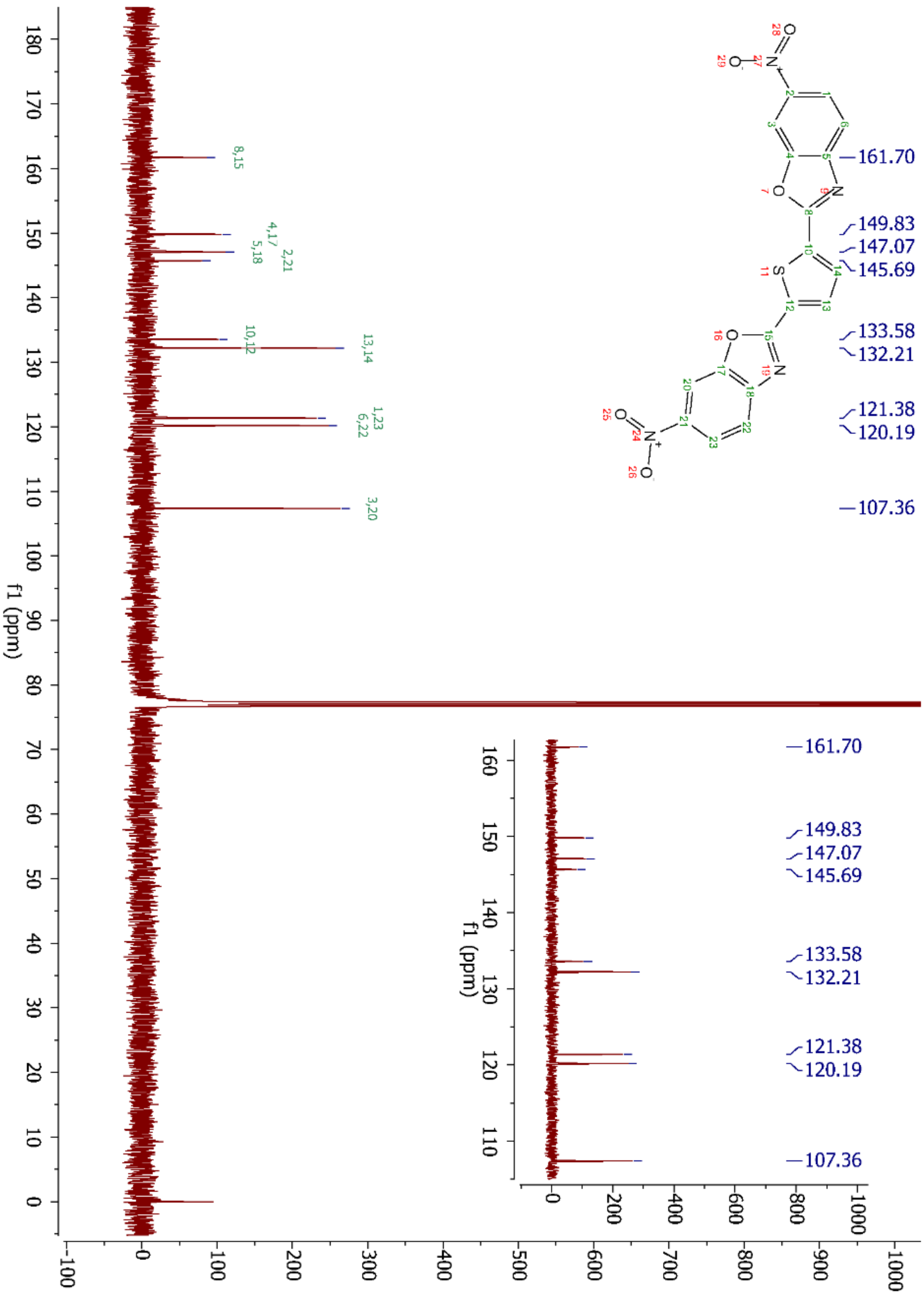

Figure S4: ^{13}C-NMR of BBTN in $CDCl_3$

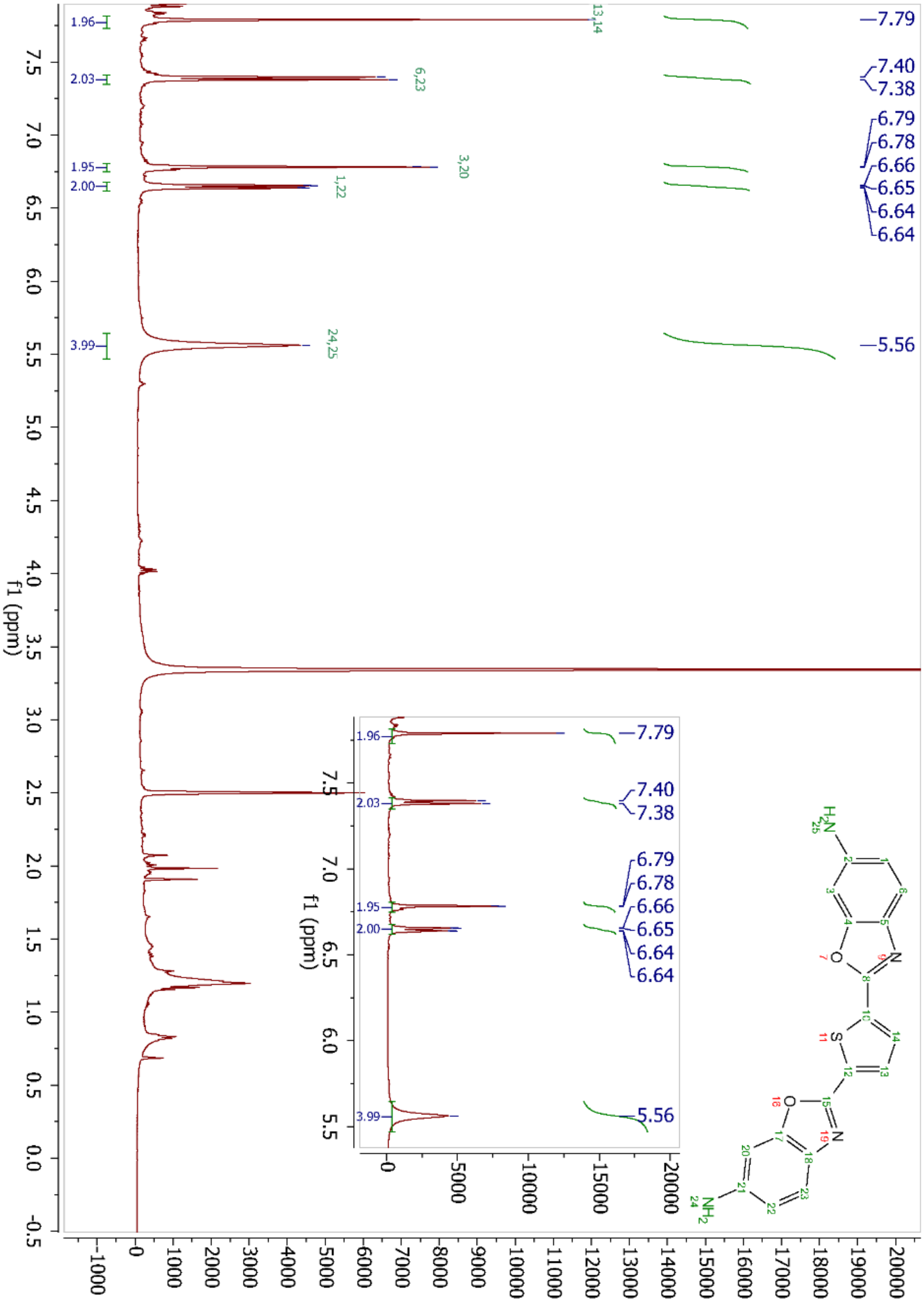

Figure S5: ^{1}H-NMR of BBTA in d_6-DMSO

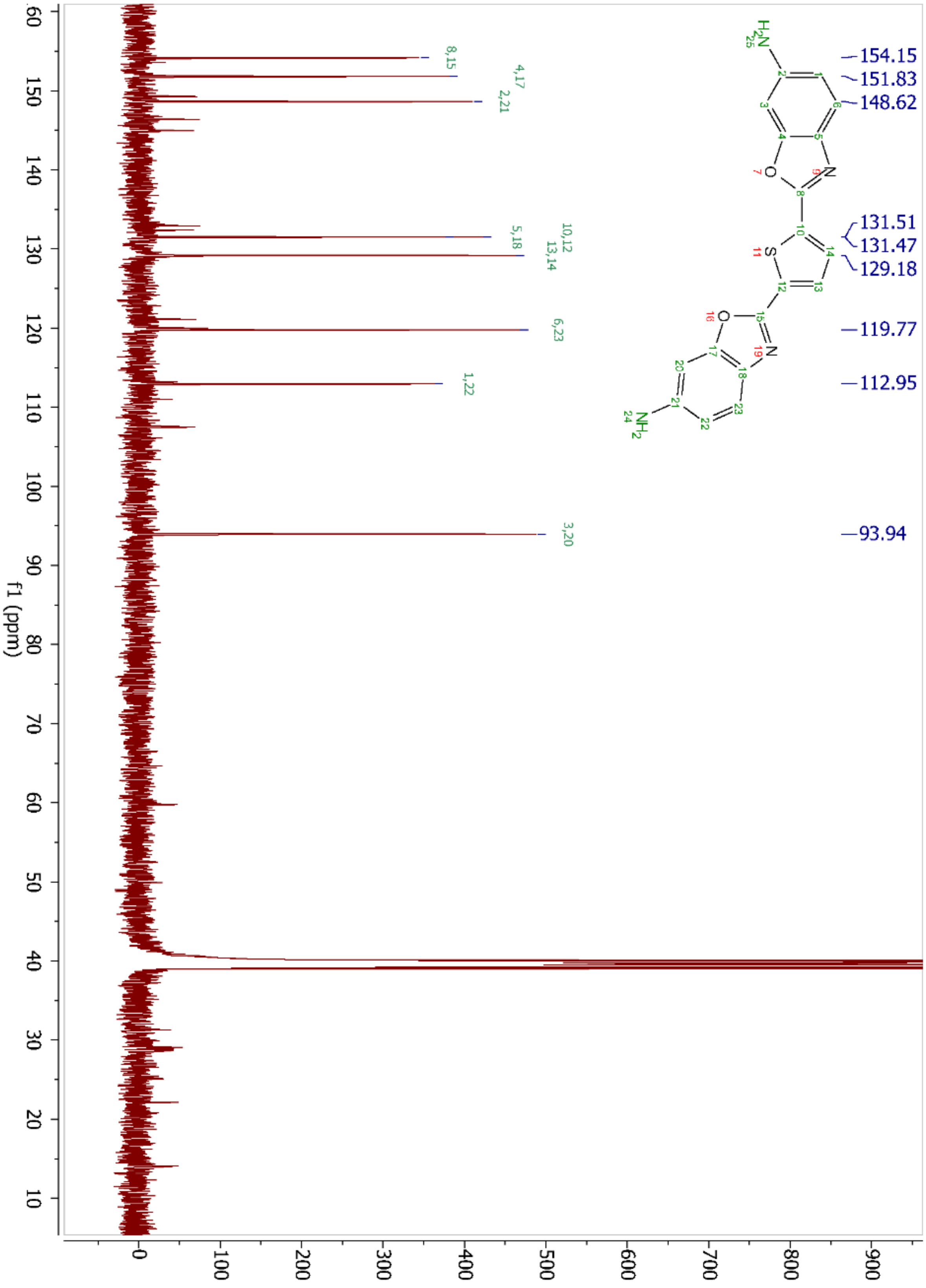

Figure S6: ^{13}C-NMR of BBTA in d_6-DMSO

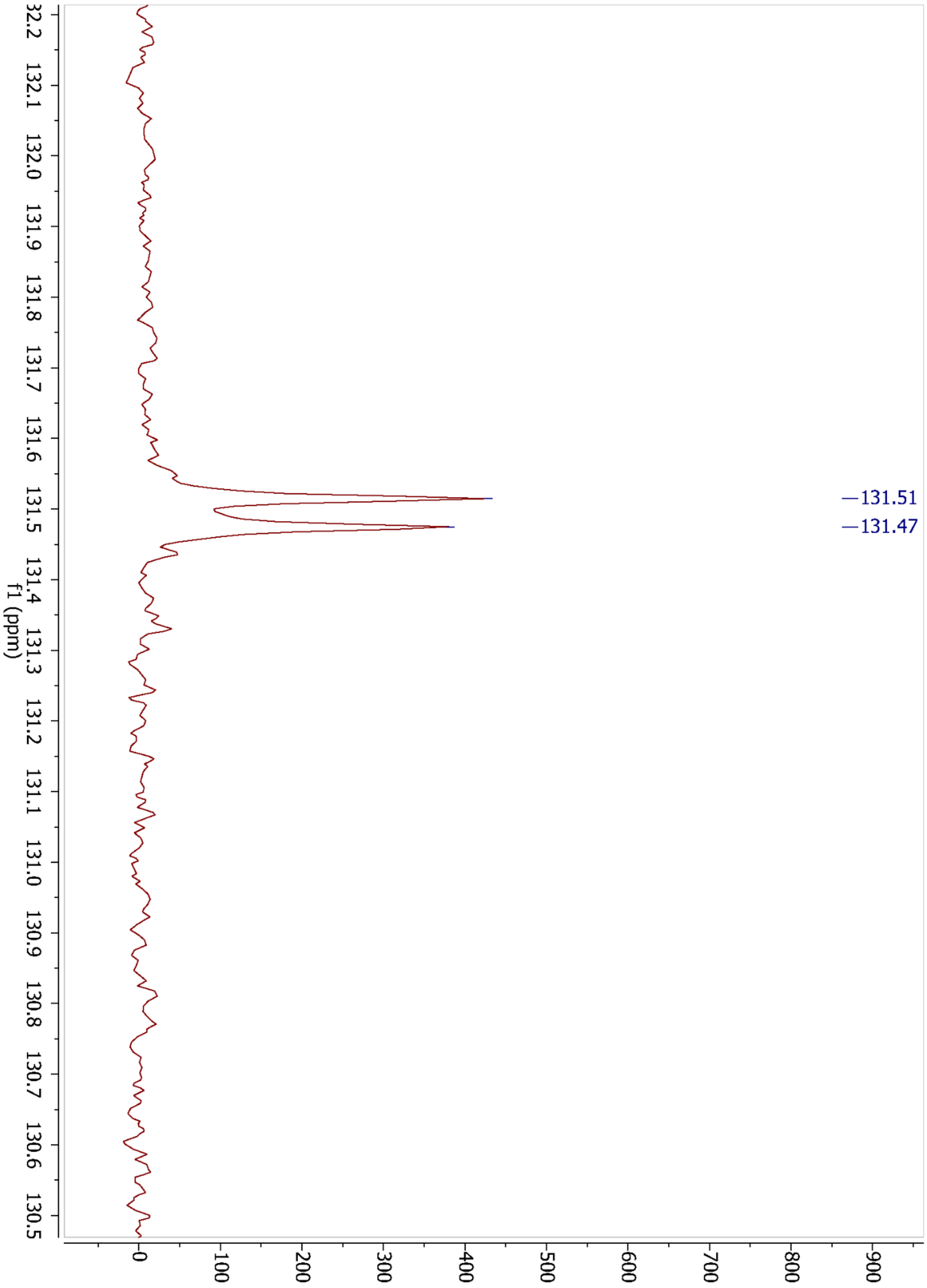

Figure S7: Zoom of the ^{13}C-NMR of BBTA in d_6-DMSO from 132 to 130.5 ppm.

4. Synthesis of molecule 7

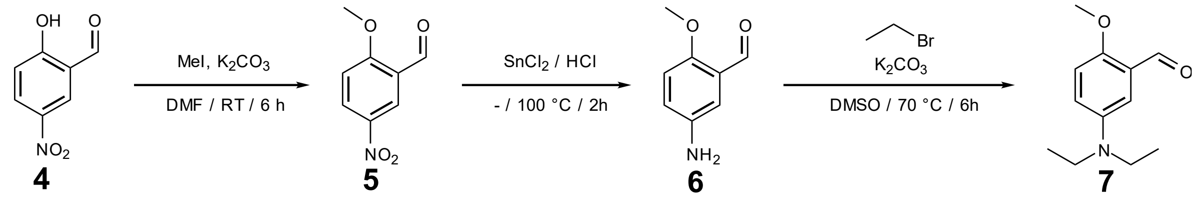

The synthesis of molecule 7 was conducted according to literature [1].

Synthesis of **5**

To a suspension of 5-nitrosalicylaldehyde (4, 1.53 g, 9.15 mmol) and K_2CO_3 (3.95 g, 28.6 mmol) in DMF (15 mL), methyl iodide (12.37 g, 87.1 mmol) was added dropwise. The reaction mixture was stirred for 12 h at room temperature. Then the residue was poured in ice water (100 mL) and the product precipitated. This suspension was filtered off and the residue was washed with water (3 x 25 mL). Finally, the residue was purified by column chromatography on silica gel.

Yield (1.588 g, 96 %) **5** as a colourless solid. R_F: 0.35 (cyclohexane/ ethyl acetate 1:1)

^{1}H-NMR (500 MHz, $CDCl_3$): δ = 10.46 (s, 1H), 8.70 (d, *J* = 2.9 Hz, 1H), 8.45 (dd, *J* = 9.2, 2.9 Hz, 1H), 7.14 (d, *J* = 9.2 Hz, 1H), 4.09 (s, 3H) ppm

^{13}C-NMR (125 MHz, $CDCl_3$): δ = 187.53, 165.54, 141.66, 130.68, 124.67, 112.24, 56.73 ppm

Synthesis of **7**

2-methoxy-5-nitrobenzaldehyde (5, 1.48 g, 8.19 mmol) and stannous chloride dihydrate (7.57 g, 39.9 mmol) were placed in a 50 mL round bottom flask and hydrochloric acid 37 % (9 mL) was added. The reaction mixture (RM) was heated to 100 °C, turning from light yellow to dark orange, and left at this temperature for 3 h. Subsequently, the RM was allowed to cool to room temperature and adjusted to pH 14 with sodium hydroxide solution 30 % (40 mL). The suspension obtained was diluted with water (50 mL) and filtered through a sieve. The crude product 6 obtained was dried overnight in a vacuum drying oven at 40 °C.

The crude product (6) was dissolved in DMSO (25 mL) and K_2CO_3 (12.48 g, 90.3 mmol), KOH (1.63 g, 29.1 mmol) and bromoethane (15.10 g, 138.6 mmol) were added. The RM was warmed to 50 °C and left at this temperature for 16 h. The RM was then poured into water. Subsequently, the RM was added to water (150 mL) and extracted with DCM (3 x 60 mL). The combined organic phases were dehydrated over sodium sulphate and filtered off over cotton wool. The solvent was removed with a rotary evaporator at a bath

temperature of 45 °C at reduced pressure. The crude product was purified by NP column chromatography.

Yield (743 mg, 43 %) **7** as an orange oil. R_F: 0.62 (cyclohexane/ethyl acetate 1:1)

^{1}H-NMR (500 MHz, $CDCl_3$): δ = 10.33 (s, 1H), 7.11 – 7.02 (m, 2H), 6.94 (d, *J* = 3.1 Hz, 1H), 3.83 (s, 3H), 3.28 (q, *J* = 7.0 Hz, 4H), 1.04 (t, *J* = 7.0, 6H) ppm.

^{13}C-NMR (125 MHz, $CDCl_3$): δ = 190.14, 154.00, 142.56, 125.13, 121.21, 113.41, 111.15, 56.20, 44.75, 12.41 ppm

5. Stokes shift of 7 in different solvents

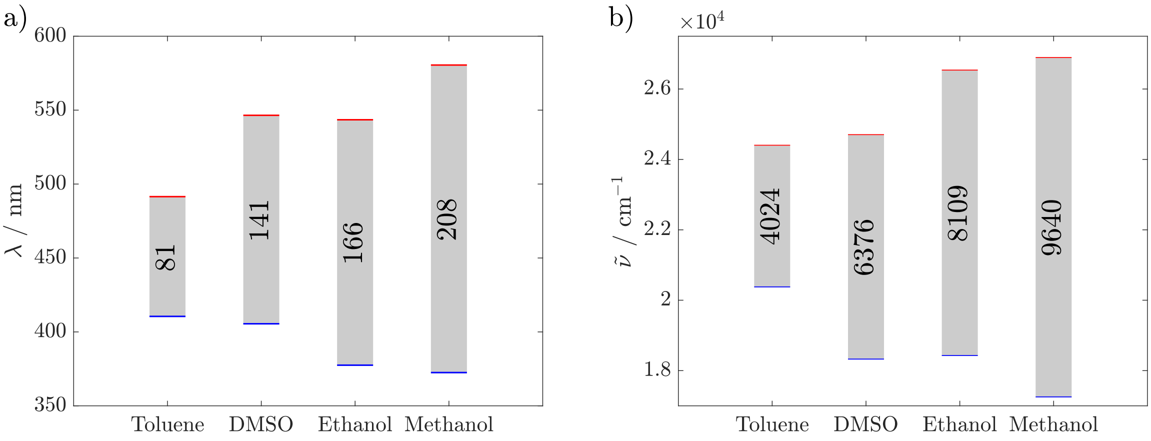

Figure S8: Stokes shift of **7** (66 µg·mL^{-1}) in a) wavelength and b) wavenumber in different solvents. The maxima, blue for excitation and red for emission, are highlighted. The Stokes shift is shown as a grey bar. The exact excitation and emission wavelength maxima are listed in Tab. S5 and S6.

Table S5: Data for Figure S8a. Molecule 7 in different solvents with the excitation and emission maximum and the corresponding Stokes shift.

Solvent	λ_{ex}^{max} / nm	λ_{em}^{max} / nm	Stokes shift / nm
Toluene	410	491	81
DMSO	405	546	141
Ethanol	377	543	166
Methanol	372	580	208

Table S6: Data for Figure S8b. Molecule 7 in different solvents with the excitation and emission maximum and the corresponding Stokes shift.

Solvent	$\tilde{\nu}_{ex}^{max}$ / cm^{-1}	$\tilde{\nu}_{em}^{max}$ / cm^{-1}	Stokes shift / cm^{-1}
Toluene	24390	20367	4024
DMSO	24691	18315	6376
Ethanol	26525	18416	8109
Methanol	26881	17241	9640

6. 3D fluorescence spectra of 7 in solution, impregnated and intercalated

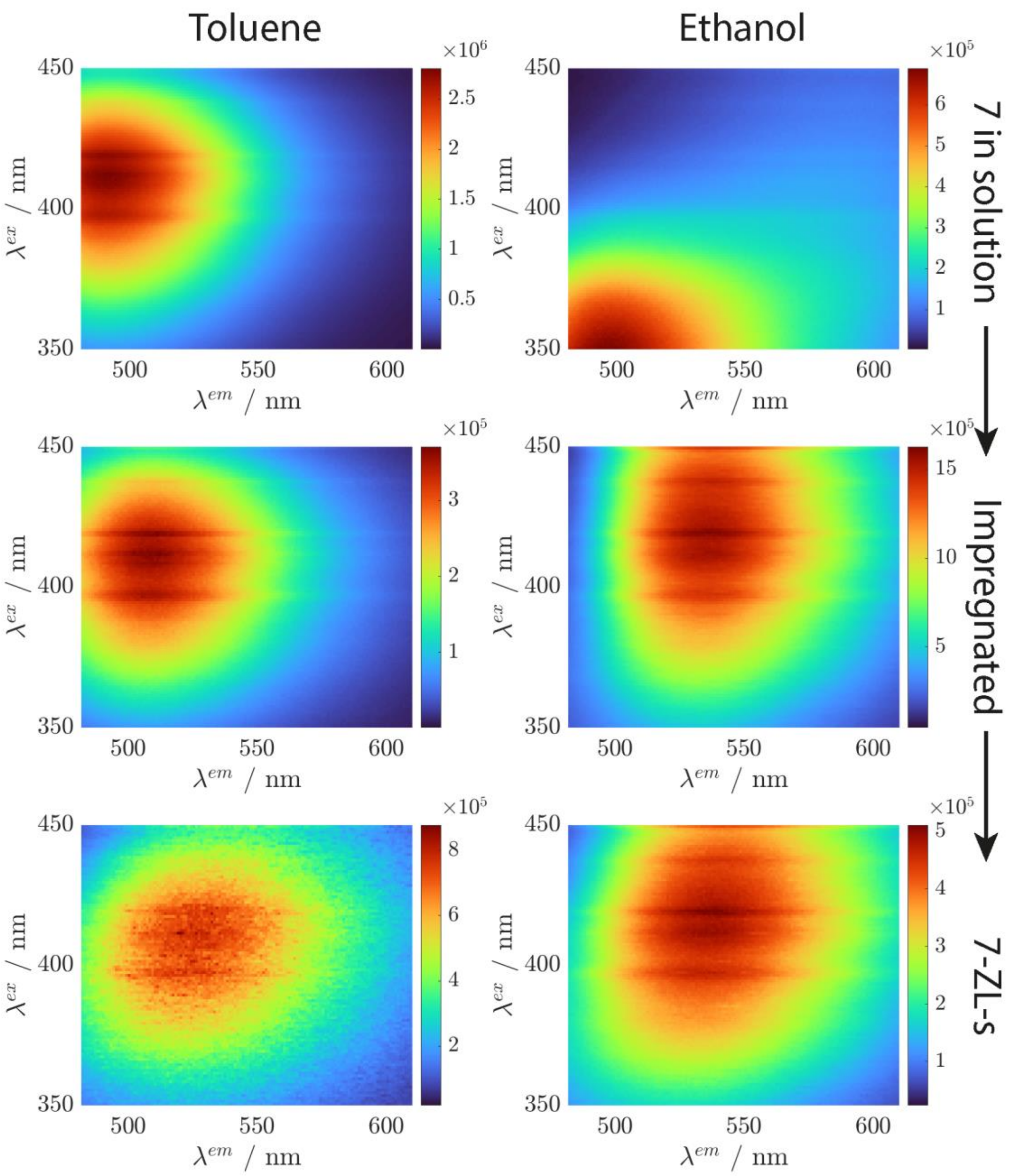

Figure S9: 3D fluorescence spectra measured in toluene (left column) and in ethanol (right column). From top to bottom: **7** in solution (66 µg·mL^{-1}), **7** on the external surface of ZL (Impregnated, 333 µg·mL^{-1}), and **7**-ZL-s (333 µg·mL^{-1}). The excitation wavelength is plotted on the y-axis and the emission wavelength is plotted on the x-axis.

7. Stokes shift of 7 after intercalation

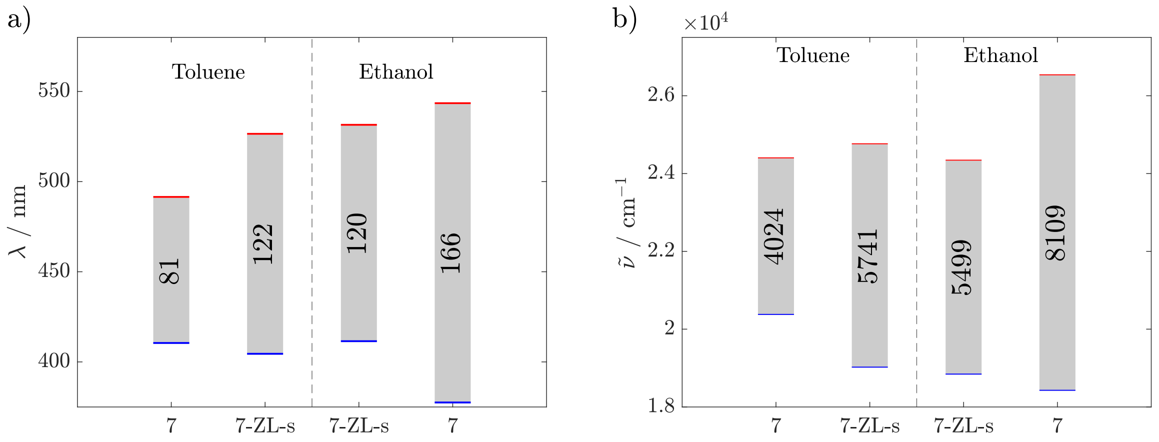

Figure S10: Stokes shift of **7** (66 µg·mL^{-1}) and **7**-ZL-s (333 µg·mL^{-1}) in a) wavelength and b) wavenumber in toluene and ethanol. The maxima, blue for excitation and red for emission, are highlighted. The Stokes shift is shown as a grey bar. The exact excitation and emission wavelength maxima are listed in Tab. S7 and S8.

Table S7: Data for Figure S10a. 7 and 7-ZL-s in different solvents with the excitation and emission maximum and the corresponding Stokes shift.

Analyte	Solvent	λ_{ex}^{max} / nm	λ_{em}^{max} / nm	Stokes shift / nm
7	Toluene	410	491	81
7-ZL-s	Toluene	404	526	122
7-ZL-s	Ethanol	411	531	120
7	Ethanol	377	543	166

Table S8: Data for Figure S10b. 7 and 7-ZL-s in different solvents with the excitation and emission maximum and the corresponding Stokes shift.

Analyte	Solvent	$\tilde{\nu}_{ex}^{max}$ / cm^{-1}	$\tilde{\nu}_{em}^{max}$ / cm^{-1}	Stokes shift / cm^{-1}
7	Toluene	24390	20367	4024
7-ZL-s	Toluene	24752	19011	5741
7-ZL-s	Ethanol	24331	18832	5499
7	Ethanol	26525	18416	8109

8. NMR of the products in the synthesis of 7

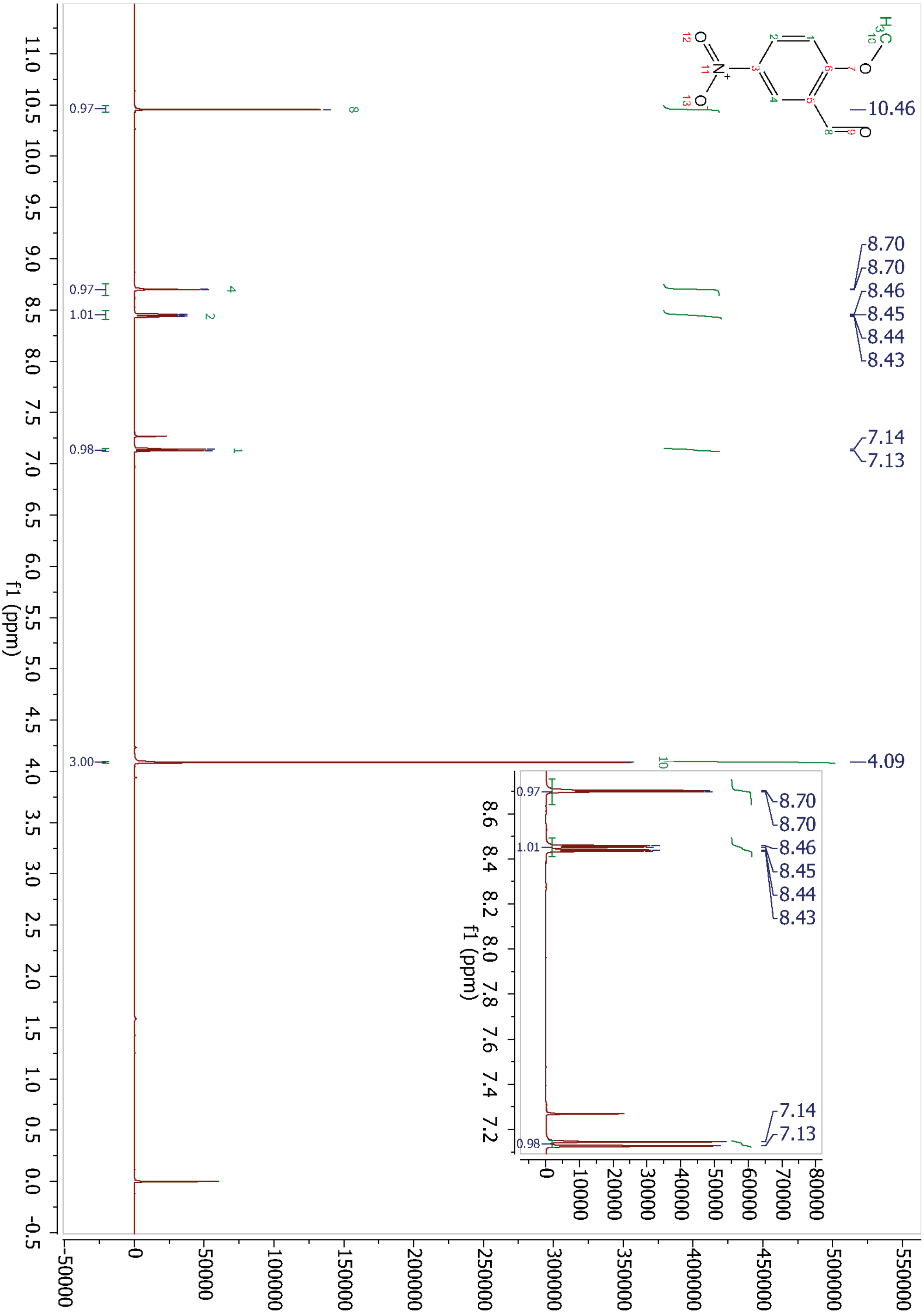

Figure S11 ^{1}H-NMR of **5** in $CDCl_3$.

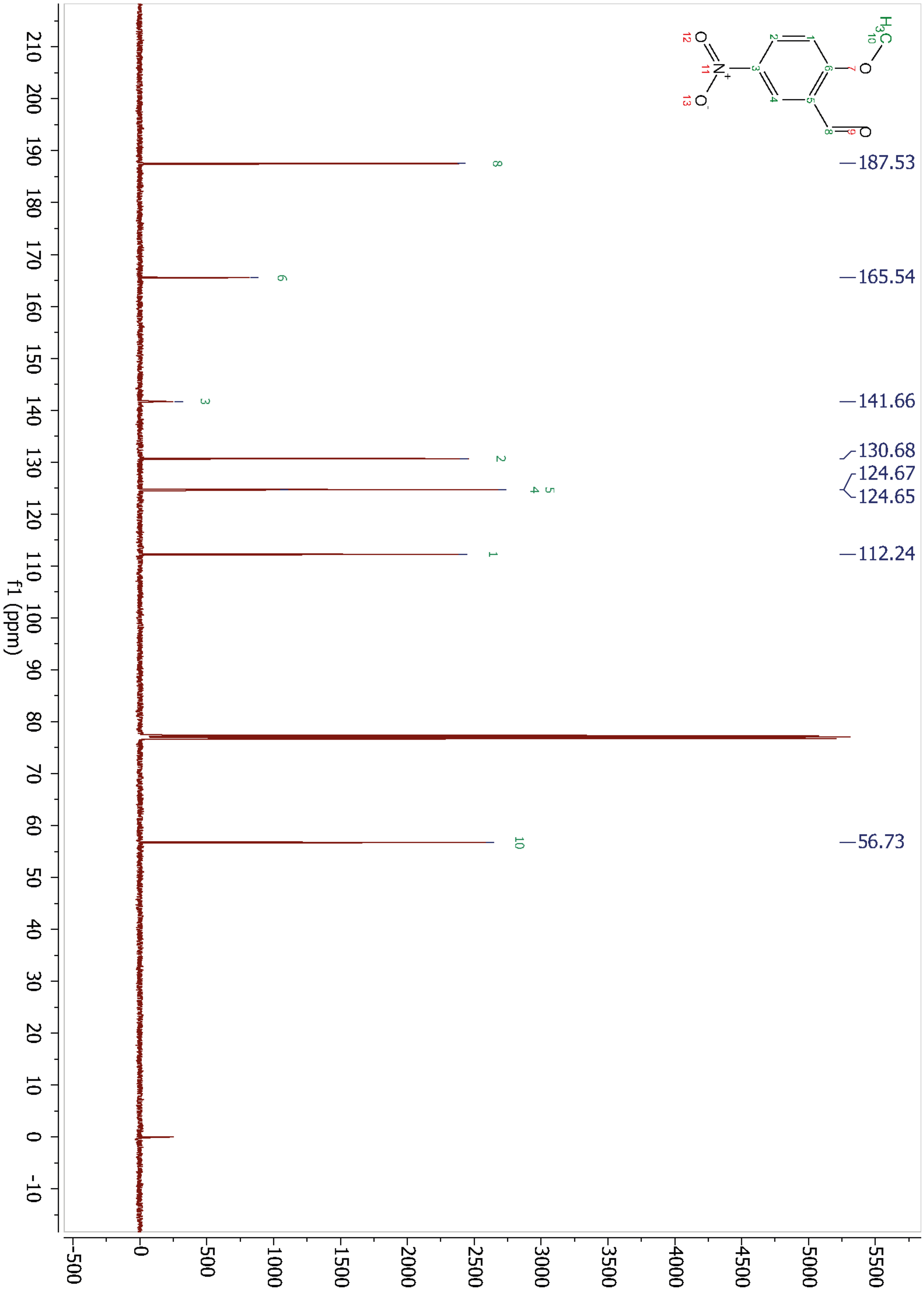

Figure S12: ^{13}C-NMR of **5** in $CDCl_3$.

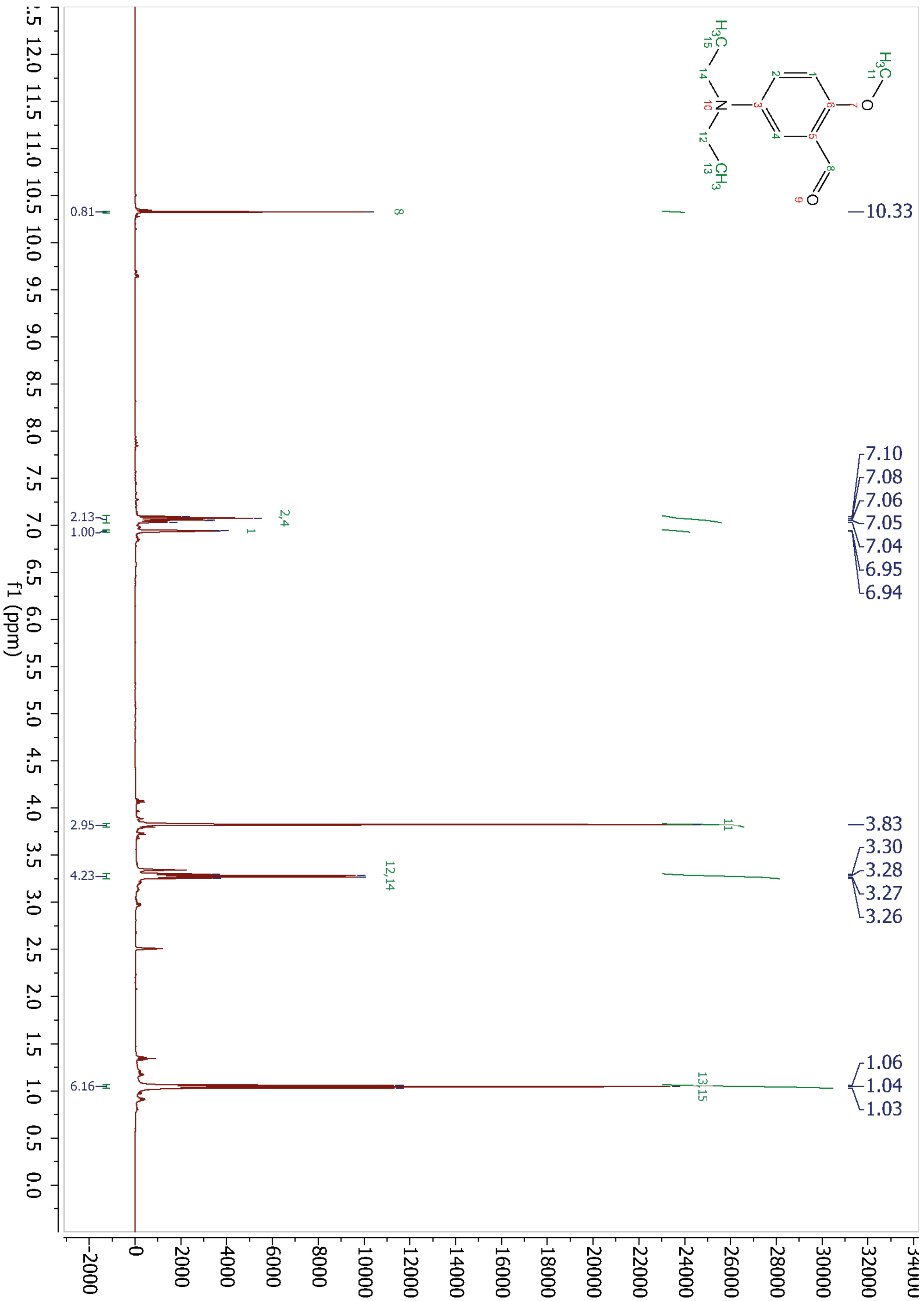

Figure S11: ^{1}H-NMR of **7** in d_6-DMSO.

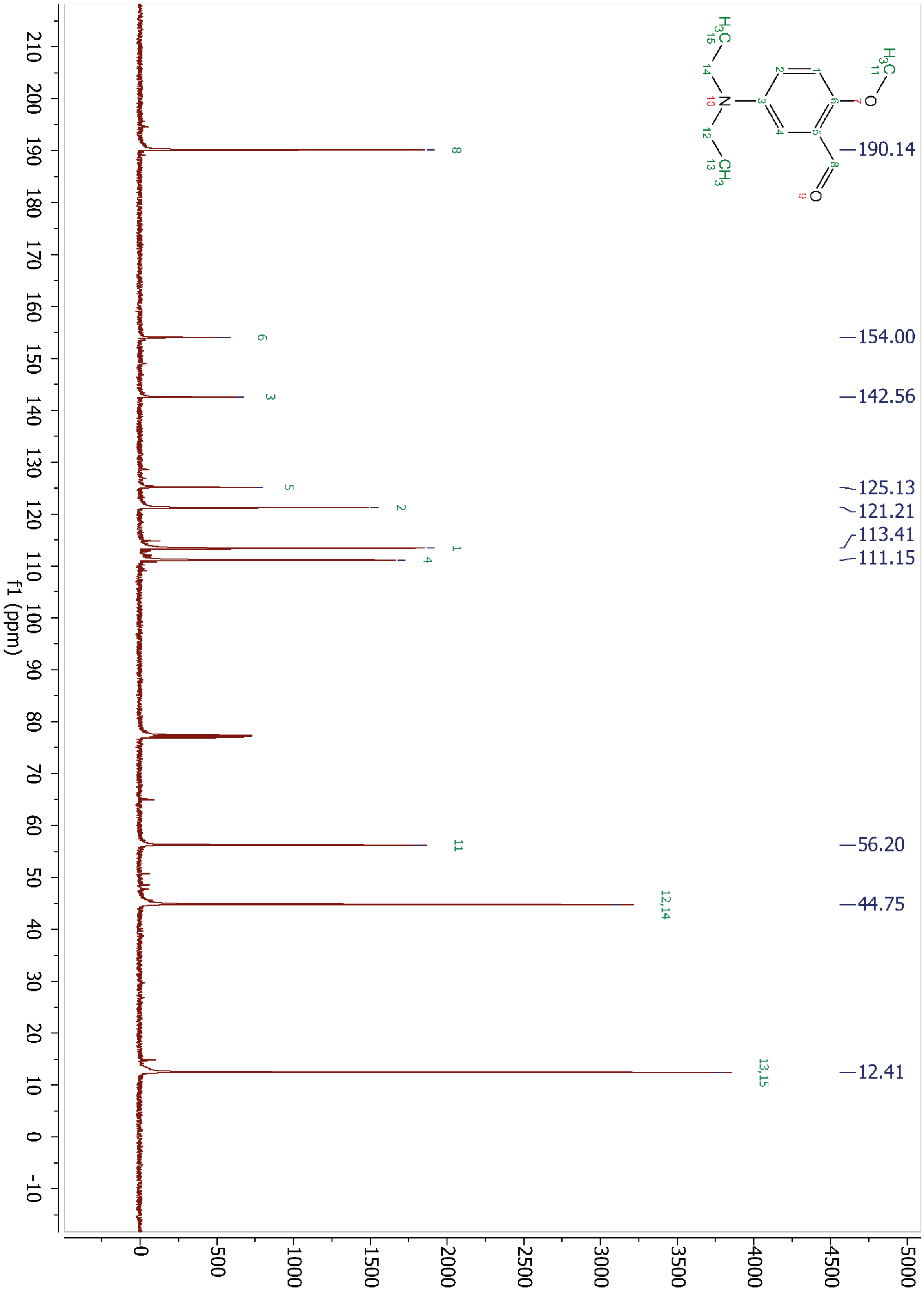

Figure S12: ^{13}C-NMR of **7** in d_6-DMSO.

9. Reference

1. Mandal, M.; Chatterjee, T.; Das, A.; Mandal, S.; Sen, A.; Ta, M.; Mandal, P.K. Meta-Fluors—A Unique Way To Create a 200 Da Ultrasmall Fluorophore Emitting in Red with Intense Stokes/Solvatochromic Shift: Imaging Subcellular Nanopolarity in Live Stem Cells. *J. Phys. Chem. C* **2019**, *123*, 24786–24792, doi:10.1021/acs.jpcc.9b08524.
